# Reliability, validity and distribution of the Spanish female sexual function index in women with relapsing multiple sclerosis

**DOI:** 10.1186/s12905-023-02811-4

**Published:** 2023-12-11

**Authors:** Sara Gil-Perotin, Salma Reddam, Cristina González-Mingot, Anna Gil-Sánchez, Inés González-Suarez, Silvia Peralta, Patricia Escrivá, Lucas Barea-Moya, Beatriz Sánchez-Sánchez

**Affiliations:** 1 Research group in Immunotherapy and Biomodels for Autoimmunity, Health Research Institute, Hospital Universitario y Politécnico La Fe, Valencia, Spain; 2 Multiple Sclerosis Unit, Neurology Department, Hospital Universitario y Politécnico La Fe, Valencia, Spain; 3 CIBER, Instituto de Salud Carlos III, Madrid, Spain; 4 Multiple Sclerosis Unit, Hospital Arnau de Vilanova, Lleida, Spain; 5 Multiple Sclerosis Unit, Hospital Álvaro Cunqueiro, Vigo, Spain; 6 Sexual and Reproductive Health Unit, Centro de Salud Trinitat, Valencia, Spain; 7 Physiotherapy Department, Faculty of Medicine and Health Sciences, University of Alcalá, Physiotherapy in Women’s Health (FPSM) Research Group, Madrid, Spain

**Keywords:** Female sexual function index, Sexual health, Factor analysis, Multiple sclerosis, Psychometric validation, Sexual dysfunction

## Abstract

**Background:**

The Female Sexual Function Index (FSFI) is a widely recognized tool for assessing sexual dysfunction (SD). However, its validation for Spanish women suffering from multiple sclerosis (MS) has not yet been conducted.

**Aim:**

The study aimed to examine the psychometric properties of the 19-item Spanish version of the FSFI (svFSFI) in women with relapsing MS.

**Method:**

A total of 137 women with relapsing MS from three Spanish centers participated in the study and completed the svFSFI. The psychometric properties of the questionnaire were evaluated. The prevalence of SD in the study cohort was determined, and its association with clinical and sociodemographic variables was analyzed using bi- and multivariate regression analyses.

**Results:**

The svFSFI demonstrated excellent test-retest reliability and substantial-to-excellent internal consistency in the context of relapsing MS. There was significant convergent validity in the intercorrelations of domains. Discriminant validity showed differences in SD between women with high and low neurological disability, as measured by the Expanded Disability Status Scale (EDSS) scores. An exploratory factor analysis indicated a five-factor structure for the svFSFI. The prevalence of SD in the MS cohort was found to be 42.6%, with the ‘desire’ and ‘arousal’ domains being the most affected. Factors such as EDSS score, fatigue, depression, and having a stable partner were found to influence the total svFSFI score.

**Conclusion:**

The study validates the svFSFI as a reliable and valid instrument for evaluating sexual dysfunction in Spanish women with MS.

## Background

The World Health Organization recognizes sexual health as a comprehensive state of well-being concerning sexuality, encompassing physical, emotional, mental, and social aspects [1]. The compromise of sexual health can lead to the onset of sexual dysfunction (SD), a condition that impacts nearly 40–50% of women [2, 3], and is characterized by clinically significant alterations in the ability to engage in sexual activity or experience sexual pleasure for at least 6 months, unless induced by medication [4]. SD is particularly prevalent in individuals with multiple sclerosis (MS), impacting their quality of life significantly [5–7]. The causes of SD are multifaceted, involving physical, neurological, psychological, and social factors [8, 9]. In women with MS, the prevalence of SD ranges from 40 to 70% [10, 11] a variation that may be attributed to the challenges in thoroughly evaluating sexual health during outpatient visits.

Research by the Consortium of Multiple Sclerosis Centers indicates that in MS consultations, symptoms like depression, anxiety, sleep, and pain are frequently evaluated (80%), but SD is assessed in only half of the cases, often through a general question, and in only 4 out of 24 cases using a specific tool [12]. Women with MS and SD commonly experience genital sensory alterations, vaginal dryness, orgasmic dysfunction, decreased libido, dyspareunia, and, though rare, hypersexuality. Genital sensory loss significantly contributes to arousal and orgasm dysfunction. The aetiology of SD in women with MS is complex, involving anatomical, physiological, biological, and psychological aspects. A detailed history and thorough examination are crucial for diagnosing SD, with a focus on the aspect that most significantly affects the woman psychologically. This includes gathering comprehensive information about her sexuality, orientation, partner relationship quality, treatment expectations, and the chronological and severity assessment of symptoms. Factors such as increased disability, pain, disease duration, and concurrent depression are linked to the sexual well-being of women with MS [13], highlighting the importance of routinely assessing sexual function in these patients during follow-up appointments.

The Female Sexual Functioning Index (FSFI) [14] is a widely used patient-reported outcome measure (PROM) for evaluating female SD. Developed by Rosen et al., it is a brief, multidimensional self-report questionnaire with 19 items assessing six factors of sexual function. The FSFI has been validated in more than 20 languages, included Spanish across different populations. The svFSFI, a Spanish adaptation of the Female Sexual Function Index, has undergone validation in Spain among women experiencing pelvic floor disorders [15]. However, this validation has not yet been extended to include women diagnosed with MS.

The present study aimed to test the psychometric properties of svFSFI in women with relapsing MS and to offer insights into the prevalence and potential contributing factors of SD in this specific population.

## Method

### Subjects and procedure

This study, a multicenter cross-sectional observational research, was conducted over a 2-year period from April 2021 to April 2023. It involved the recruitment of participants from specialized Multiple Sclerosis (MS) Units at three Spanish hospitals: Hospital Universitario y Politécnico La Fe, Hospital Álvaro Cunqueiro in Vigo, and Hospital Arnau de Vilanova in Lleida. The study focused on women who met specific inclusion criteria: a diagnosis of relapsing-remitting MS [16], to have sexual activity within the previous 4 weeks, being over 18 years of age and being able to read and understand Spanish. As exclusion criteria: confirmed progressive disease independent of relapses, having reached the menopause or mental incapacity to properly fill in the questionnaire.

The sample size for this study was determined using the guidelines provided by Terwee et al. for psychometric validation [17]. The authors recommend a ratio of at least 4 individuals per item on the questionnaire, with a minimum of 100 subjects to ensure statistical reliability and validity. Additionally, an anticipated loss-to-follow-up rate of 10% was factored into the calculation. Given that the Female Sexual Function Index (FSFI) comprises 19 items, the study aimed to enroll a minimum of 110 women to meet these criteria and ensure a robust and reliable analysis of the data. During the baseline interview, clinicians registered sociodemographic and clinical information on forms. This included recording the hospital where each participant was treated, their age, the duration of their disease since diagnosis, any history of childbirth, and details about their partnership and professional status. The assessment also covered neurological disability and whether the participant was undergoing disease-modifying therapy (DMT) or symptomatic central nervous system (CNS) agents. Additional information gathered included a history of myelitis, symptoms of sphincter dysfunction such as voiding urgency, urinary retention, and urinary and/or fecal incontinence.

### Measures

At the beginning of the study, during the baseline interview, all participating women filled out several questionnaires. ﻿After a detailed explanation of the questionnaires, most of the patients completed a pseudonymized online form for clinical purposes outside the outpatient clinic, with the exemption of those who lacked internet devices or web navigation skills that filled up a paper form after the clinical visit.

The FSFI is a comprehensive self-report questionnaire designed to evaluate various aspects of sexual function in women. It consists of 19 items that cover six key sexual domains over the preceding 4 weeks: desire (assessed by items 1 and 2), arousal (items 3 through 6), lubrication (items 7 to 10), orgasm (items 11 to 13), satisfaction (items 14 to 16), and pain (items 17 to 19). Each question is scored on a scale ranging from 0 or 1 to 5, where higher scores represent better sexual function and less pain experienced during sexual activity. To calculate the total score for each domain, a validated correction factor is applied, capping the maximum score for each domain at 6. Consequently, the highest possible total FSFI score is 36, indicating optimal sexual function, while the lowest score is 2, reflecting more severe SD [14]. In this study, a subset of 21 women completed the FSFI on two occasions, with an interval of 6 to 8 weeks between assessments, to evaluate the test-retest reliability of the instrument. This time frame was chosen to ensure that the participants’ symptoms remained stable, while also being sufficiently long to prevent recall of their initial responses. During this period, there was no reported disease activity, nor were there any changes in the participants’ treatments. To assess the discriminant validity of the FSFI, the women were categorized based on their level of disability, as measured by the Expanded Disability Status Scale (EDSS) [18]. They were divided into two groups: those with an EDSS score of less than 3 and those with a score of 3 or higher. This categorization allowed for the examination of differences in sexual function between women with varying degrees of disability.

The Modified Fatigue Impact Scale (MFIS) is a specialized tool designed to measure the impact of fatigue in individuals with MS. This 21-item scale evaluates the effects of fatigue over the past 4 weeks across three distinct functional domains: cognitive, physical, and psychosocial [19, 20]. The total MFIS score is calculated by summing the scores from each of these domains, with the overall score ranging from 0 to 84. Higher scores on the MFIS indicate a greater level of fatigue. In clinical practice and research, a threshold score of 38 is commonly used to differentiate between patients who are fatigued and those who are not [21].

The Beck Depression Inventory-II (BDI-II) is a widely recognized and extensively used tool for assessing the severity of depression [22]. This instrument focuses on the most recent 2-week period and comprises 21 items. Each item is scored on a scale from 0 to 3, with the total score reflecting the severity of depressive symptoms. The BDI-II employs standardized cut-off points to categorize the level of depression: scores from 0 to 13 indicate minimal depression, 14 to 19 suggest mild depression, 20 to 28 are indicative of moderate depression, and scores ranging from 29 to 63 denote severe depression. These cut-offs provide a useful guide for clinicians and researchers in interpreting the severity of depression in individuals.

### Psychometric validation

The psychometric validation of the svFSFI in women with MS involved assessing its reliability, validity, interpretability, and feasibility.

- Reliability: This was evaluated in two ways:

- Internal Consistency: measures how closely related the items are within the questionnaire. Cronbach’s alpha was calculated as an indicator, with a value of 0.7 considered acceptable [17].
- Test-Retest Reliability: examines the instrument’s ability to consistently measure a sample over time. The intraclass correlation coefficient (ICC) was used, employing a 2-way random effects, single measures, and absolute agreement model [17, 23]. Measurement error was determined using the standard error of measurement (SEM_agreement_), calculated with the formula SD x √1 - ICC. The smallest detectable change (SDC) was calculated at both the individual and group levels [24].

- Construct Validity: This was evaluated through:

- Structural Validity: assesses how well each item relates to the hypothesized domain, using exploratory factor analysis (EFA) with Varimax orthogonal rotation [25]. Bartlett’s sphericity test and the Kaiser-Meyer-Olkin (KMO) normalization test were used to assess suitability, with values above 0.6 indicating good suitability [26]. The number of factors was determined based on the Kaiser eigenvalue criterion and sedimentation graph evaluation.
- Convergent Construct Validity: measures how scores relate logically to other variables, using Spearman correlations between questionnaire dimensions and the total score. Effect sizes were calculated to determine the magnitude of differences [27].

- Discriminant Construct Validity: This aspect of the study focused on evaluating the scale’s capacity to distinguish between different participant groups based on anticipated variations in scores. The key clinical variable used for this differentiation was the degree of disability, which was assessed using the EDSS with a threshold value set at 3.0. To compare scores between groups, the Wilcoxon-Mann-Whitney test was employed.
- Interpretability and Feasibility: Although not psychometric measures, these characteristics were still considered [28]. Interpretability was assessed using the percentage of unanswered individual items and the ceiling-floor effect. Feasibility was assessed by calculating the average completion time [17].

### Assessment of prevalence and association with demographic and clinical variables

For sample description, categorical variables were expressed as counts and percentages, while continuous variables were expressed as mean or median. The FSFI cut-off value of 26.55 established by Wiegel et al. was used to diagnose SD [29]. Associations between FSFI scores and registered variables were analyzed using non-parametric tests (Mann-Whitney test for two groups) or Kruskal Wallis test for more than two groups and Spearman’s test. Bivariate and multivariate linear regression analyses were used to adjust for influencing covariates, with multiple comparisons and correlations adjusted using the Benjamini-Hochberg procedure to reduce the false discovery rate (FDR).

### Patient and public involvement

Our primary objective in conducting this study was to assess the psychometric properties of the FSFI as a tool for comprehensive evaluation of sexual function in women with MS. Our goal was to identify SD and the specific affected domains, with the aim of enhancing the quality of care for these women. The potential impact of our work on the general public is that it may raise awareness, provide better support, and ultimately lead to improved healthcare outcomes for women living with MS.

## Results

### Participants

The study recruited a total of 137 women from three hospitals in Spain between April 2021 and April 2023. The distribution of participants was as follows: Hospital Universitario y Politécnico La Fe (Valencia) (*n* = 70), Hospital Arnau de Villanova (Lleida) (*n* = 28), and Hospital Alvaro Cunqueiro (Vigo) (*n* = 39). The demographic and clinical characteristics of these participants are detailed in Table 1. On average, the women were 36.4 years old. A significant majority of the cohort, 84%, had a low level of disability, as indicated by scores of less than 3 on the EDSS.

**Table 1:** Clinical and demographic characteristics of women with MS included in the study (*n* = 137)

Age (years), X (SD)	38.3 (7.2)
Disease duration (years), X (SD)	8.3 (6.0)
History of childbirth, *n* (%)	52 (49.1)
Stable partner, *n* (%)	91 (75.2)
Professional status: active, *n* (%)	94 (75.2)
EDSS (median, p25 – p75)	2 (1–2.5)
- EDSS < 3, *n* (%)	115 (83.9)
- EDSS ≥3, *n* (%)	22 (16.1)
Patients on DMT	
- No treatment	20 (14.6)
- First line, *n* (%)	38 (27.7)
- heDMT, *n* (%)	79 (57.7)
Myelitis, *n* (%)	85 (62.5)
- Cervical, *n* (%)	41 (53.9)
- Dorsal, *n* (%)	9 (11.8)
- > 1 level, *n* (%)	26 (34.2)
- Diffuse, *n* (%)	0
Sphincter symptoms, *n* (%)	53 (38.7)
Referred fatigue, *n* (%)	51 (37.8)
MFIS (*n* = 58), X (SD)	20.4 (18.3)
Depression on specific treatment	18 (13.2)
BDI-II (*n* = 54), X (SD)	23.5 (19.6)
Women on CNS depressants, *n* (%)	32 (23.5)

*SD* standard deviation, *EDSS* Expanded Disability Status Scale, *DMTT* disease-modifying therapy, *heDMT* high efficacy DMT, *MFIS* Modified Fatigue Impact Scale, *BDI-II* Beck Depression Inventory-II

### Psychometric validation

#### Reliability

 The svFSFI demonstrated excellent internal consistency, as evidenced by a high Cronbach’s alpha coefficient of 0.98 (0.97–0.98) (Table 2). Test-retest reliability was also robust, with an ICC of 0.96 (95% CI: 0.91–0.98) for the total score (Table 3).

**Table 2:** Internal consistency reliability for dimensions and total FSFI (*n* = 137)

Domain	Cronbach’s Alpha
Desire	0.885
Arousal	0.947
Lubrication	0.965
Orgasm	0.943
Satisfaction	0.905
Pain	0.958
Total FSFI	0.977

*FSFI* Female Sexual Function Index

**Table 3:** Test-retest reliability for dimensions and total FSFI (*n* = 21)

	Test, mean (SD)	Retest, mean (SD)	SEM_agreement_	SDC_ind_	SDC_group_	ICC (95%CI)
Desire	3.4 (1.3)	3.5 (1.3)	0.51	1.41	0.31	0.845 (0.66–0.93)
Arousal	4.3 (1.5)	4.3 (1.5)	0.47	1.31	0.29	0.901 (0.77–0.96)
Lubrication	4.5 (1.5)	4.6 (1.5)	0.34	0.94	0.21	0.948 (0.88–0.98)
Orgasm	4.9 (1.4)	4.8 (1.4)	0.44	1.22	0.26	0.9 (0.77–0.96)
Satisfaction	4.9 (1.1)	4.9 (1.2)	0.30	0.86	0.19	0.928 (0.83–0.97)
Pain	5.2 (1.5)	5.0 (1.5)	0.34	0.95	0.21	0.948 (0.87–0.98)
Total FSFI	27.3 (7.3)	27.2 (7.3)	1.44	3.99	0.87	0.96 (0.91–0.98)

*FSFI* Female Sexual Function Index, *SD* Standard Deviation, *SEM* Standard Error of Measurement, *SDCind* Smallest Detectable Change (individual level), *SDC group* Smallest Detectable Change (group level), *ICC* Intraclass Correlation Coefficient, *CI* Confidence Interval

#### Validity

**Fig. 1:**
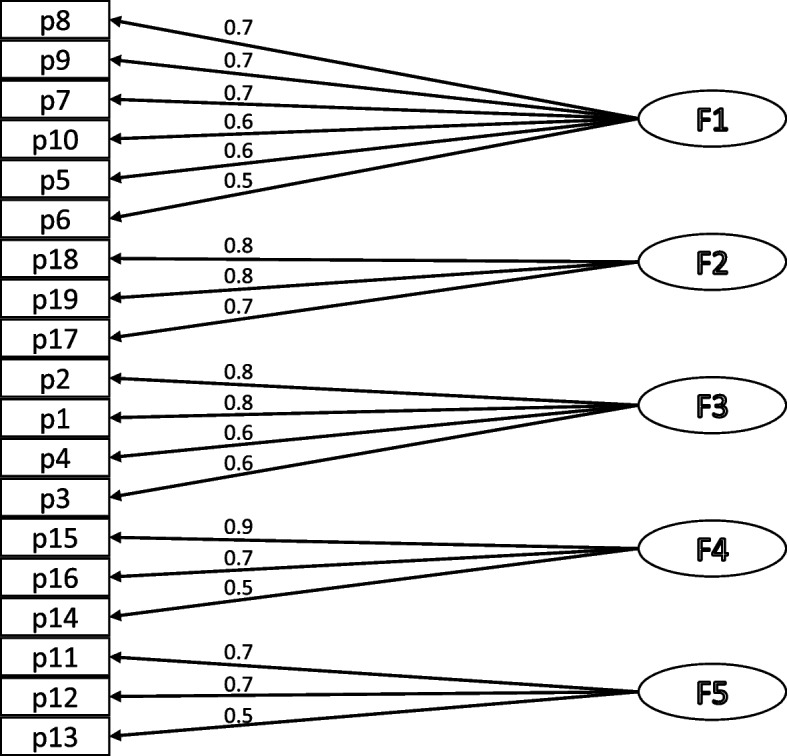
FSFI five-factor model with item factor loadings

The factor structure of the svFSFI in our cohort was confirmed through a KMO test, which yielded a value of 0.95, and a Bartlett sphericity test that significantly rejected the null hypothesis (*χ*2 = 3478.4; *p* < 0.001). Exploratory Factor Analysis (EFA) identified five factors using a minimum eigenvalue of 1.0 for factor extraction, explaining a cumulative variance of 85% (Table 4 and Fig. 1). The ‘Arousal’ items were loaded into two distinct factors associated with desire and lubrication, while other items corresponded to the remaining four dimensions initially described by Rosen et al. [14]. All items clustered predictably with high factor loadings, affirming the factorial validity of the FSFI in women with MS.

- Convergent construct validity indicated strong correlations within all domains and with the total FSFI score. The domains with the highest intercorrelations were arousal with lubrication, satisfaction, orgasm, and pain; lubrication with orgasm and pain; and orgasm with pain (Table 5).
- In terms of discriminant construct validity, women with an EDSS score < 3 reported significantly higher FSFI scores (26.2) compared to those with an EDSS score ≥ 3 (8.7), with no differences observed in the domain. These differences might be influenced by factors such as longer disease duration and higher age in the latter group (Table 6).

**Table 4:** Factor analysis of the FSFI (*n* = 137)

*Items*	*Domain: item*	F1	F2	F3	F4	F5
Item 1	Desire: frequency	0.15	0.10	0.80	0.18	0.15
Item 2	Desire: level	0.16	0.17	0.85	0.20	0.07
Item 3	Arousal: frequency	0.37	0.42	0.56	0.25	0.28
Item 4	Arousal: level	0.47	0.31	0.57	0.30	0.31
Item 5	Arousal: confidence	0.56	0.38	0.48	0.22	0.34
Item 6	Arousal: satisfaction	0.53	0.35	0.37	0.29	0.49
Item 7	Lubrication: frequency	0.73	0.33	0.34	0.28	0.27
Item 8	Lubrication: difficulty	0.74	0.39	0.17	0.26	0.29
Item 9	Lubrication: frequency of maintaining	0.73	0.35	0.26	0.28	0.31
Item 10	Lubrication: difficulty in maintaining	0.63	0.46	0.24	0.21	0.38
Item 11	Orgasm: frequency	0.43	0.31	0.25	0.25	0.69
Item 12	Orgasm: difficulty	0.42	0.40	0.23	0.28	0.67
Item 13	Orgasm: satisfaction	0.41	0.39	0.17	0.36	0.55
Item 14	Satisfaction: with closeness with partner	0.45	0.41	0.28	0.52	0.32
Item 15	Satisfaction: with sexual relationship	0.24	0.23	0.26	0.86	0.19
Item 16	Satisfaction: with overall sex life	0.24	0.23	0.31	0.72	0.21
Item 17	Pain: frequency during vaginal penetration	0.35	0.73	0.23	0.23	0.21
Item 18	Pain: frequency following vaginal penetration	0.32	0.83	0.21	0.25	0.27
Item 19	Pain: level during or following vaginal penetration	0.38	0.75	0.23	0.27	0.28
Eigenvalue		14.55	2.38	1.84	1.65	1.37
% of explained variance		22	19	17	14	13

*FSFI* Female Sexual Function Index

**Table 5:** FSFI Domains intercorrelations (Spearman rho)

TOTAL	Desire/arousal	Lubrication/arousal	Orgasm	Satisfaction	Pain	Global
Desire/arousal	1					
Lubrication/arousal	0.785^a^	1				
Orgasm	0.699^a^	0.876^a^	1			
Satisfaction	0.720^a^	0.775^a^	0.763 ^a^	1		
Pain	0.683^a^	0.820^a^	0.779 ^a^	0.715^a^	1	
Total FSFI	0.868^a^	0.955^a^	0.915 ^a^	0.869 ^a^	0.884 ^a^	1

^a^*P* < 0.05; FSFI, Female Sexual Function Index

**Table 6:** Discriminant construct validity: correlation of FSFI dimensions depending on EDSS

	EDSS < 3 (*n* = 104)				EDSS > 3 (*n* = 21)				*p*–value
	*Mean*	*SD*	*Median*	*p25 – p75*	*Mean*	*SD*	*Median*	*p25 – p75*	
Desire	3.5	1.2	3.6	2.4–4.2	3.1	1.3	2.7	2.4–4.65	0.183
Arousal	4.2	1.7	4.8	3.3–5.4	3.1	1.7	3.6	2.2–4.4	0.001
Lubrication	4.5	1.9	5.1	4.1–6	3.5	2.2	4.2	2.8–5.3	0.008
Orgasm	4.3	1.9	5.2	3.6–5.6	3.2	2.1	3.6	2–5.1	0.004
Satisfaction	4.6	1.6	4.8	4–6	3.6	1.6	3.8	2.4–4.8	0.002
Pain	4.9	1.8	6.0	4.8–6	3.8	2.1	4.2	3.3–5.8	0.004
Total FSFI	26.2	8.7	28.9	23.6–32.3	20.3	9.9	24.1	15.7–26.9	0.002

*FSFI* Female Sexual Function Index, *EDSS* Expanded Disability Status Scale

#### Interpretability

All participants completed the questionnaires, with less than 15% of women scoring the lowest (*n* = 1) or highest (*n* = 2) possible total FSFI scores, indicating an absence of ceiling or floor effects. The average time to complete the questionnaire was 5.1 min.

### svFSFI score assessment in women with MS: prevalence and influencing variables for SD

The overall mean svFSFI score in the study was 25.3, with a standard deviation of 9.1, and the median score was 28.0 (interquartile range: 22.4–31.9). The scores across different domains are detailed in Table 7. Utilizing the established cut-off value of 26.55 for SD, 57 women in the cohort were classified as having SD, resulting in a prevalence rate of 42.6% in the studied group. After adjusting for covariates and the FDR, a significant correlation was observed between the total FSFI scores and baseline disability (as measured by the EDSS scale), as well as scores on the MFIS and BDI-II questionnaires (Table 8).

**Table 7:** Scores dimensions and total FSFI (*n* = 137)

*Domains*	*X*	*SD*	*Median*	*p25 – p75*
Desire (1.2–6.0)	3.5	1.2	3.6	2.4–4.2
Arousal (0–6.0)	4.1	1.7	4.5	3.3–5.4
Lubrication (0–6.0)	4.4	2.0	5.1	3.6–6.0
Orgasm (0–6.0)	4.1	2.0	4.8	3.2–5.6
Satisfaction (0.8–6.0)	4.5	1.6	4.8	3.6–6.0
Pain (0–6.0)	4.8	1.9	5.6	4.4–6.0
Total FSFI (2.0–36)	25.3	9.1	28	22.4–31.9

*FSFI* Female Sexual Function Index, *X* mean, *SD* standard deviation

**Table 8:** Correlation between clinical variables and total FSFI (*n* = 137)

Variable	Spearman Rho	*P*- value	Adjusted *P*- value *
Baseline EDSS	- 0.303	0.000	0.002*
Baseline age	- 0.124	0.148	0.222
Baseline disease duration	- 0.037	0.666	0.666
Childbirths	0.092	0.448	0.538
MFIS	- 0.316	0.015	0.032*
BDI-II	- 0.323	0.016	0.032*

*FSFI* Female Sexual Function Index, *EDSS* Expanded Disability Status Score, *MFIS* Modified Fatigue Impact Scale, *BDI-II* Beck Depression Inventory-II

(*) Significant after Benjamini-Hochberg test

The analysis revealed that the median total svFSFI score was notably lower in women who were being treated for depression (*p* = 0.002), those reporting fatigue (*p* = 0.001), those with sphincter symptoms (*p* = 0.034), and in participants taking CNS depressant medications (*p* < 0.0001). Interestingly, neither a history of myelitis nor the use of high efficacy DMT were associated with significant changes in svFSFI scores. Conversely, higher total svFSFI scores were observed among women who had a stable partner (*p* = 0.018) and those who were employed (*p* = 0.0001). In the multivariate linear regression analysis, the factors that significantly influenced SD in the cohort were the BDI-II score, MFIS score, and having a stable partner.

## Discussion

This study focused on evaluating the psychometric properties of the Spanish version of the FSFI in a cohort of women with relapsing MS. The findings robustly support the reliability and validity of the svFSFI for use in this particular cohort. Notably, the study revealed a high prevalence of SD among these women and demonstrated the complex interconnections between SD and factors such as the level of disability, relationship status, depression, and fatigue.

The exploratory factor analysis (EFA) revealed a five-factor structure, which differed from the initially proposed six-factor model [14, 30]. Notably, the arousal items were found to be encompassed within two distinct factors: desire and lubrication. This deviation from the original structure might be attributed to the complex interplay between cognitive and physiological aspects of sexual response, as suggested by recent revisions [31]. Regarding structural validity, in the present study, the EFA identified five factors that explained 85% of the total variance, including the domains of desire/arousal, arousal/lubrication, orgasm, satisfaction, and pain. This indicated that the arousal items were integrated into two factors: item 3 and 4 in desire/arousal and item 5 and 6 in lubrication/arousal, eliminating the arousal factor as an independent entity. Although Rosen and colleagues initially proposed a five-factor solution for the original FSFI, they extended the FSFI structure to six domains due to their consideration of a clinical distinction between desire and ‘arousal.’ A systematic review of measurement properties of the FSFI, according to COnsensus-based Standards for the selection of health Measurement INstruments (COSMIN) guidelines, indicates inconsistent structural validity of the FSFI in distinct countries and populations [32]. While the authors observed strong criterion validity supporting the scale’s use in screening for SD, they reported conflicting evidence for some measurement properties, recommending structural factor analysis for each study cohort before its use. The authors concluded that there was more evidence against than in favor of the hypothesized 6-factor structure. Most studies support a five-factor solution through confirmatory factor analysis (CFA) and principal component analysis (PCA) [32, 33], in which ‘desire’ and ‘arousal’ are interdependent, aligning with recent revisions made in the DSM-5, where ‘desire’ and ‘arousal’ were merged into ‘female sexual interest/arousal disorder’ [31]. ‘Desire’ could be considered a cognitive component within the ‘arousal’ category, and the distinction between ‘desire’ and ‘arousal’ can be challenging in some women, leading to cross-loading in factor analysis [34, 35]. Other validations with PCAs have resulted in distinct factor structures in different subgroups or nationalities, possibly due to cultural differences, varying motivations for sex in women with or without arousal disorder [36], or the extensive overlap in sexual disorders [37–39]. These differences may also stem from varying sample characteristics or measurement properties of the instrument used in different studies. We conclude that the 5-factor solution is a valid structure for using the Spanish FSFI in our cohort of women with MS. Nevertheless, larger studies or validation by external groups are warranted.

But why use FSFI instead of a specific questionnaire for MS, such as the Multiple Sclerosis Intimacy and Sexuality Questionnaire (MSISQ-19) [40]? Compared to MSISQ-19, FSFI offers a comprehensive evaluation of multiple domains of female sexual function, enabling the identification of specific affected areas and guiding the selection of appropriate interventions, which was our initial objective in this study [41]. It also facilitates comparisons between cohorts of women with MS and other neurological patients with different pathologies. FSFI has been widely validated across various populations and languages. Additionally, FSFI assesses SD over the previous 4 weeks, making it more sensitive to recent changes. Importantly, it enables comparisons of SD between individuals with and without MS, thus providing insights into prevalence among distinct populations. We believe that both scales are complementary, and we recommend conducting a psychometric validation of the Spanish version of MSISQ-19 in the female population with MS, still lacking to our knowledge.

The prevalence of SD in our cohort was 42.6%. This prevalence is similar to that reported in the general female population (40–50%) by other studies [2, 3] and falls within the lower range for women with MS, as studies have reported SD rates ranging from 40 to 80% [42, 43]. This may be because our cohort consists primarily of young premenopausal women with low levels of disability and relapsing-remitting forms of MS. The inclusion of women with progressive forms of MS may increase the prevalence of SD, and further investigations are planned. However, based on our clinical experience, the frequency of sexual intercourse in the most affected population was low due to disability. Regarding the affected domains, in our cohort, SD was primarily related to ‘desire’ and ‘arousal,’ with ‘pain’ being the least affected domain. In other studies, the most frequent symptoms varied, including ‘dyspareunia’ and ‘anorgasmia’ [44], ‘anorgasmia’ and decreased vaginal lubrication [45], ‘alteration of sensory function in the genital area’ and ‘decreased libido’ in women with advanced MS [46], and ‘fatigue,’ ‘decreased sensations,’ and ‘decreased libido’ [47]. These variations in symptoms may be influenced by the degree of disability in the study cohort. We found a relationship between SD and the degree of neurological disability, fatigue, and sphincter dysfunction. Having a stable partner appeared to be a protective factor. Fatigue, depression, and anxiety are common symptoms among individuals with MS and can significantly impact their sexual function. These relationships with SD have been shown to be common and independent of other factors studied [48–53]. Psychoeducational interventions and medical approaches for anxiety and depressive symptoms have shown a positive impact on SD [54, 55]. Age may also be a predictive factor for SD among women with MS, with older individuals potentially experiencing more difficulties [49, 56, 57]. Neurological disability has also been related to SD. As in our sample, the EDSS score, using different cut-off values, has been consistently related to SD in previous reports [56, 58–60]. Specific sphincter symptoms, such as urinary incontinence or bowel dysfunction, could also negatively impact sexual function in individuals with MS [61, 62], although we could not confirm this association. These symptoms can lead to discomfort or embarrassment during sexual activity, resulting in a decrease in sexual desire and satisfaction. One study specifically reported overactive bladder as an independent factor of SD in women with MS [43]. For those women suffering from sphincter symptoms, therapeutic interventions have shown an improvement in SD after non-blinded, single-arm trials [63–65]. The potential negative effect of CNS depressants on SD in our population was observed but did not reach statistical significance after correcting for multiple comparisons.

A limitation of this study is the imbalance in the distribution of patients with EDSS scores below and above 3. This disparity may have impacted the outcomes of the discriminant validity analysis, potentially influencing both the prevalence and the most reported symptoms. The study did not consider thyroid function as a variable, which is often present in women with MS and has been recognized as a factor affecting sexual function. Furthermore, the study did not assess the questionnaire’s responsiveness to intervention, a critical psychometric attribute that evaluates the tool’s ability to detect changes in response to treatment. Despite these limitations, the study’s strengths are noteworthy. It represents the first validation of the svFSFI in women with MS. The research benefits from a relatively large sample size, encompassing multiple centers, and the comprehensive completion of the questionnaire by all participants.

## Conclusion

In summary, this study assessed the psychometric properties of the svFSFI in women with relapsing MS. The prevalence of SD in this cohort was 42.6%, primarily related to ‘desire’ and ‘arousal.’ Factors associated with SD included neurological disability, fatigue, depression, anxiety, and sphincter dysfunction, while having a stable partner appeared to be protective. The use of CNS depressants showed a potential negative effect on SD. The study also highlighted the importance of addressing SD in clinical practice to improve the quality of care provided to women with MS. The incorporation of the svFSFI in clinical settings promises to improve the quality of sexual health care for women with MS, ensuring their concerns are both recognized and appropriately managed.

## Data Availability

The datasets used and/or analysed during the current study are available from the corresponding author on reasonable request.

## Abbreviations

BDI-II: Beck depression inventory-II
CFA: Confirmatory factor analysis
CNS: Central nervous system
COSMIN: COnsensus-based Standards for the selection of health Measurement INstruments
DMT: Disease modifying therapies
EDSS: Expanded disability severity scale
EFA: Exploratory factor analysis
FDR: False discovery rate
FSFI: Female Sexual Function Index
heDMT: High efficacy DMT
ICC: Intraclass correlation coefficient
KMO: Kaiser-Meyer-Olkin
MFIS: Modified Fatigue Impact Scale
MS: Multiple sclerosis
MSISQ-19: Multiple Sclerosis Intimacy and Sexuality Questionnaire
PCA: Principal component analysis
PROM: Patient-reported outcome measures
SD: Sexual dysfunction
SDC: Smallest detectable change
SEM: Standard Error of the Mean
svFSFI: Spanish version FSFI
